# Physical activity and mammographic density in a cohort of postmenopausal Norwegian women; a cross-sectional study

**DOI:** 10.1186/2193-1801-1-75

**Published:** 2012-12-21

**Authors:** Samera Azeem Qureshi, Merete Ellingjord-Dale, Solveig Hofvind, Anna H Wu, Giske Ursin

**Affiliations:** 1 Department of Nutrition, Institute of Basic Medical Sciences, University of Oslo, P.Box 1110, Blindern, Oslo, Norway; 2 Cancer Registry of Norway, Majorstuen, P.O.Box 5313, Oslo, 0304 Norway; 3 Department of Preventive Medicine, University of Southern California, Los Angeles, California USA

**Keywords:** Epidemiology, Mammographic density, Physical activity, Breast cancer, Screening

## Abstract

Mammographic density (MD) is a strong risk factor for breast cancer and may represent a useful intermediate marker for breast cancer risk. Physical activity (PA) is known to be associated with a reduced risk of breast cancer. If PA is associated with MD then this would be useful for breast cancer prevention studies. MD was assessed on digitized mammograms using a computer assisted method (Madena) in 2218 postmenopausal women. A questionnaire assessed PA, by asking about the duration and intensity of light, moderate, strenuous PA/week. We used multivariate linear regression models to estimate least square means of percent MD by total and intensity of PA with adjustment for confounders. The mean age (± s.d) was 58.4 (±5.3) and mean BMI was 24.6 (±4.6). We observed a statistically significant inverse association between total PA and MD in the over-weight (BMI = 25.0-29.9) women, where mean MD among women with highest activity (>360 mins/week) was 12.6% (95%CI; 11.2%-14.0%), while among women with no activity it was 15.9% (95 CI; 13.6%-18.2%, p for trend = 0.04). There was no association in the other BMI strata. MD was 12.1% (11.2%-13.0%) in the highest group (> 180 mins/week) of moderate/strenuous activity and in the no activity group 14.8% (14.2%-15.5%, p for trend = 0.001) in the over-weight women. There was no association between light PA and MD in all women combined or in any other BMI strata. We found some evidence of an inverse association between PA and MD among overweight women.

## Introduction

Physical activity is one of the few modifiable lifestyle factor, which may play an important role in the prevention of breast cancer (World Cancer Research Fund/American Institute for Cancer Research 2007; WCRF/AICR Systematic Literature Review Continuous Update Report 2008). According to the International Agency for Research on Cancer (IARC), approximately 25% of cancer cases worldwide occur as a result of obesity and sedentary lifestyle (IARC Working Group 2002). Regular (or high) physical activity has been associated with a reduced risk of breast cancer in epidemiological studies (Thune et al. 1997; McTiernan et al. 2003; McTiernan 2010; Carmichael et al. 2010; World Cancer Research Fund/American Institute for Cancer Research 2007; Irwin et al. 2006; Bernstein et al. 1994; Friedenreich 2001; Peters et al. 2009; Lahmann et al. 2007; George et al. 2010; Friedenreich 2011; Lynch et al. 2011; WCRF/AICR Systematic Literature Review Continuous Update Report 2008), but details including the type of activity or the amount and intensity of activity that is needed to confer protection remains to be determined.

High mammographic density is positively associated with risk of developing breast cancer for both pre- and postmenopausal women (McCormack & Dos Santos 2006). Mammographic density is usually expressed as a percentage: the area on the mammogram that is radiologically dense divided by the whole breast area. The risk of breast cancer is 4 to 6 times in women having a mammographic density > 75% as compared to women having very low or no density (McCormack & Dos Santos 2006).

Mammographic density has been shown to be influenced by sex hormone levels (Boyd et al. 2002a). Reproductive factors such as nulliparity and late age at first birth which are positively associated with breast cancer risk have been shown to be positively associated with high mammographic density (Gram et al. 1995). Moreover, combined hormone therapy has been associated with high mammographic density (Greendale et al. 2003; McTiernan et al. 2005). Physical activity may protect against breast cancer through hormonal mechanisms (Friedenreich & Cust 2008), either directly through influencing circulating hormones or protein levels, or indirectly by reducing body mass resulting in lower levels of circulating sex hormones (van Gils et al. 2009), or by increasing insulin sensitivity. Thus it is possible that physical activity exerts its effect on breast cancer through mammographic density. Further, women who are physically active are often leaner than women who are not, and body mass index (BMI) is inversely associated with mammographic density (Stone et al. 2009), thus complicating the association between physical activity and mammographic density.

Epidemiological studies on the association between physical activity and mammographic density have reported inconsistent results (Lopez et al. 2003; Monninkhof et al. 2007; Irwin et al. 2006; Masala et al. 2009; Siozon et al. 2006; Gram et al. 1999; Suijkerbuijk et al. 2006; Reeves et al. 2007; Samimi et al. 2008; Conroy et al. 2010; Peters et al. 2008). A few have reported a statistically significant inverse association between physical activity and mammographic density (Masala et al. 2009; Lopez et al. 2003; Irwin et al. 2006; Monninkhof et al. 2007). But most studies have found no evidence of an association (Peters et al. 2008; Suijkerbuijk et al. 2006; Siozon et al. 2006; Reeves et al. 2007; Samimi et al. 2008; Conroy et al. 2010; Gram et al. 1999). A number of these studies have reported a positive association between physical activity and mammographic density that attenuated after adjustments for BMI (Peters et al. 2008; Reeves et al. 2007).

In this cross-sectional study we evaluated the association between total and intensity of physical activity and mammographic density in postmenopausal Norwegian women in all women combined as well as stratified by BMI.

## Materials and methods

### Study sample

We used data from the Norwegian Breast Cancer Screening Program , a governmentally funded mammographic screening program (Hofvind 2007). Women aged 50–69 years are invited to a bilateral two-view mammogram biennially.

The methods of this mammographic density study have been described previously (Qureshi et al. 2011a). Briefly, in 2004, an informed consent form and a study questionnaire on various breast cancer risk factors were enclosed with the official Norwegian Breast Cancer Screening Program invitation letter to a random sample of 17,050 women, residing in three counties. Women were asked to bring the signed informed consent and the completed risk factor questionnaire with them to the screening examination. A total of 12,056 (71%) women attended the screening; and 7,941 (66%) returned a completed questionnaire. The questionnaire solicited answers to questions on breast cancer risk factors including menstrual and reproductive history, oral contraceptive and menopausal hormone use, family history of breast cancer, current weight and height. In addition these 7.941 women were asked whether they would be willing to complete another questionnaire on dietary intake and physical activity.

The 7,174 (90%) women who agreed were sent a 13- page questionnaire, and a total of 3,484 (49%) women returned a completed questionnaire. We requested screening mammograms from the various radiological facilities, and limited these requests to women who provided a completed dietary/physical activity questionnaire, and who had undergone screen film mammography in 2004. Among the approximately 3180 women with a completed questionnaire who had undergone screen film mammography in 2004, we were during the study period able to obtain and scan the 2004 mammograms on 2871 women. Women were excluded if they had a previous diagnosis of breast (n = 12) or ovarian (n = 5) cancer. We also excluded 291 women, in successive order, because of missing data on the following variables: women who had incomplete data on age (n = 29), weight (n = 42), height (n = 30), number of children (n = 59), education (n = 32), menopausal status (n = 62), age at menarche (n = 15), hormone use (n = 13), number of pregnancies (n = 9). A previous coding error was corrected on age for two women, resulting in two less exclusions than our previous analysis (Qureshi et al. 2011a; Qureshi et al. 2011b). We restricted this analysis to women who were assumed to be postmenopausal (six months or longer since last menstrual bleeding), at the time of screening (n = 2254). We also excluded (n = 36) women who provided no physical activity information. Finally data set in this analysis included 2218 women.

All participants signed an informed consent. The project was approved by the regional ethics committee and the Norwegian Data Inspectorate.

### Physical activity assessment

In the questionnaire women were asked to report the amount of time they usually spent on physical activity in a week. The questionnaire was modeled after the physical activity questionnaire used in the California Teachers Study (Dallal et al. 2007) and modified somewhat for Norwegian women. In the questionnaire women were asked to provide information on how much time they spent on physical activity during a week. Specifically they were asked to provide the duration of time they spent on: 1) light activities (such as walking at a slow pace, or cross-country skiing at a slow pace); 2) moderate activities (defined as activities where some effort is required and which cause somewhat increased breathing such as riding a bike at a moderate pace, swimming at a moderate pace, jogging slowly, cross-country skiing at moderate pace, dancing, golf); 3) Very strenuous activities (defined as activities that require hard work and causes substantial increased breathing such as aerobics, running or cycling fast, swimming fast, cross-country skiing at a fast pace, ball games). Women were asked to give the duration in minutes or hours/week of the three pre-defined levels of physical activity. They had to choose from eight options which were 0, < 30 minutes, 30 minutes- 1 hr, 1.5- 2 hrs, 2.5- 3.5 hrs, 4–6 hrs, 7–10 hrs and > 11 hrs per week.

### Mammographic density assessment

Total breast area and absolute mammographic density were determined using the University of Southern California Madena computer-based threshold method of assessing density, a method that has been validated and described previously (Ursin et al. 1998; Greendale et al. 2003). Left cranio-caudal mammograms were digitized using a Kodak Lumisys 85 scanner (Kodak, Rochester, New York, USA) and were then viewed on a computer screen. In brief, a trained reader first defines the total breast area using an outlining tool, and the software estimates the total number of pixels in the breast (total breast area). Next, a reader defines a region of interest in the breast that contains all the densities, but that excludes the pectoralis muscle, prominent veins, fibrous strands and other light artifacts. The reader then uses a tinting tool to apply a yellow tint to dense pixels within the region of interest that have grey levels at or above some threshold and below a pixel value of 255. The reader searches for the best threshold where all pixels within the region of interest are considered to represent mammographic densities. The software estimates the number of tinted pixels within the region of interest (absolute density). The percent mammographic density (% MD), or the fraction (%) of the breast with densities, is the ratio of absolute density to the total breast area. The density assessments were performed by GU, while the total breast areas were assessed by a research assistant trained by GU. The readers were blinded to all subject characteristics.

### Statistical analysis

As the residuals from the models satisfied the normality and homoscedasticity assumptions, we analyzed percent mammographic density as continuous variable without applying any transformation (Altman 1991). We estimated the association between physical activity and % MD by running multiple linear regression methods (Altman 1991).

We assessed the association between physical activity and % MD using women who reported no activity, defined as women who reported none to less than 30 minutes of light activity, as the reference category. In the analysis of the intensity of physical activity, we examined each activity separately, and we also modeled light, moderate and strenuous activity in the same model. We modeled light, moderate and strenuous activity in the same model; the results presented are from this model, unless stated otherwise. Tests for trend were conducted by modeling intensity of physical activity as continuous variables. The p-values are based on the ordinal value of each category of intensity of physical activity.

We also combined moderate and strenuous physical activity and created a new variable (moderate + strenuous activity) in our analysis. In this analysis we adjusted for light physical activity as well as other potential confounders in all women combined and stratified by BMI.

As indicated above women missing data on all three physical activity variables were excluded. A woman who had not answered the questions on how often she did physical activity at one of the three intensity levels, was assumed to have no activity at that intensity level, i.e. she was placed in the no activity category for that intensity level. For example, if a woman replied to the question on light physical activity and was missing on moderate and strenuous activity then it was assumed that she only did light physical activity and vice versa. We conducted analysis both with the women with such missing variables included in the no activity group as well as excluded. As the results were materially the same from these two analyses, we present the results with those with missing values included in the no activity group.

Age was defined as age at the time of mammographic screening. Both weight and height were self reported by the women. BMI was calculated by dividing weight (kilograms) by height (in meters^2^). We selected the following potential confounders *a priori* according to their presumed association with mammographic density evident from previous studies: age at mammography (years), BMI at mammography (continuous), years of education (≤ 11, 12–14, 15+), age at menarche (years), number of pregnancies (nulliparous, 1, 2, 3, 4, 5+), age at first birth among parous women (<20, 20–24, 25–29, 30+), and postmenopausal hormone therapy (never, past, current). Current alcohol intake did not alter the results, so it was not included in the final model. We did not have information on smoking, however smoking has not been strongly associated with mammographic density.

The effect of BMI on mammographic density is well established (Stone et al. 2009). In order to control for the effect of BMI on the association between physical activity and mammographic density, we tried various BMI adjustments, including adjusting for BMI quartiles (tertiles, quintiles) using more detailed BMI categories, entering them as dummy variables or as continuous variables (results not shown). We concluded that this did not necessarily capture the confounding effect at the extreme categories of BMI. We therefore also conducted analyses stratified by BMI, i.e. we examined the association between physical activity and percent mammographic density separately in women according to the World Health Organization (WHO) classification of normal weight (BMI < 25), overweight (≥ 25.0- 29.9 ), and obese (BMI ≥ 30) (WHO 1995).

Factors such as age and hormone therapy could potentially modify the association between physical activity and mammographic density. Therefore, we also tested for effect modification by age and postmenopausal therapy, by stratifying analysis by age and postmenopausal hormone therapy (never, past, current). We used χ^2^ tests for heterogeneity and trend to evaluate differences in estimates of mammographic density.

All *P* values quoted are two sided. We considered *P* values < 0.05 as statistically significant and values 0.05- 0.10 as borderline significant. All analyses were performed using the software package STATA version 11 (StataCorp. 2009. College Station, TX: StataCorp LP).

## Results

The mean age (± standard deviation) of the 2,218 participants was 58.4 years (±5.3 years) and their mean BMI was 24.6 (± 4.6). The mean physical activity as reported by the women was 288 mins/week (**±** 235 mins/week).

Normal weight women were found to be more educated and active as compared to the over- weight and obese women (Table 1). Overall there was a slight positive association between total physical activity and mammographic density in all women combined, but this was not statistically significant (p for trend = 0.65) (Table 2). This positive association was also observed in the normal weight BMI stratum. However, we found a statistically significant inverse association between total physical activity and mammographic density in overweight women. The percent mammographic density was 12.6% (11.2%- 14.0%) in the highest group (> 360 mins/week) whereas in the no activity group it was 15.9% (13.6% - 18.2%). The inverse association between total physical activity and mammographic density was also observed in the obese women, but it did not reach statistical significance.

**Table 1:** **Characteristics of all the study population and stratified by BMI**

VARIABLES	ALL	In strata of BMI		
		< 25	≥ 25-29	≥ 30
	N = 2218	N = 1235	N = 732	N = 251
	mean (± SD)	mean (± SD)	mean (± SD)	mean (± SD)
**Age (yrs)**	58.4 (**±** 5.3)	58.4 (**±** 5.1)	58.2 (**±** 5.2)	58.8 (**±** 5.2)
**Education (yrs)**	12.8 (**±** 3.3)	13.5 (**±** 3.2)	12.8 (**±** 3.2)	12.1 (**±** 3.2)
**BMI**	24.6 (**±** 4.6)	21.8 (**±** 1.8)	26.5 (**±** 1.4)	32.7 (**±** 3.4)
**Number of pregnancies**	2.5 (**±** 1.2)	2.4 (**±** 1.2)	2.5 (**±** 1.2)	2.6 (**±** 1.3)
**Age at first full-term pregnancy (yrs)**	22.1 (**±** 7.5)	22.1 (**±** 8.5)	22.1 (**±** 7.7)	21.8 (**±** 7.6)
Postmenopausal HT*	51%	54%	60%	50%
**Percent density (%)**	18.2 (**±** 15.9)	26.2 (**±** 17.7)	18.8 (**±** 14.2)	10.7 (**±** 11.5)
**Absolute density (cm**^**2**^**)**	23.5 (**±** 20.9)	26.6 (**±** 18.8)	24.8 (**±** 20.9)	19.3 (**±** 22.0)
**Physical activity (mins/week)**	288 (**±** 235)	318 (**±** 250)	266 (**±** 217)	201 (**±** 172)

*Percentage of women taking hormone therapy.

**Table 2:** Multivariate adjusted^*^mean (95%CI) of percent (%) mammographic density by duration of physical activity (mins/week) for all women and stratified by BMI

VARIABLES	ALL		In strata of BMI					
			< 25		≥ 25-29		≥ 30	
	N = 2218		N = 1235		N = 732		N = 251	
Physical_activity (mins/week)								
No activity	140	15.6 (14.0- 17.3)	58	21.8 (19.4- 24.2)	46	15.9 (13.6- 18.2)	36	8.4 (5.9- 10.9)
30- 45	129	16.2 (14.6- 17.7)	59	22.2 (20.3- 24.0)	46	15.2 (13.5- 17.0)	24	8.1 (6.3- 10.0)
46-100	280	16.7 (15.8- 17.5)	138	22.5 (21.7- 23.9)	108	14.6 (13.3- 15.8)	34	7.9 (6.5- 9.3)
101-180	408	17.8 (17.1- 18.5)	215	23.0 (22.0- 23.8)	134	13.9 (13.0- 14.8)	59	7.7 (6.5- 9.0)
181-360	630	18.5 (18.0- 19.0)	353	23.2 (22.2- 24.1)	221	13.2 (12.2- 14.3)	56	7.5 (6.0- 9.0)
> 360	631	19.9 (19.4- 20.4)	412	23.6 (22.4- 24.8)	177	12.6 (11.2- 14.0)	42	7.3 (5.2- 9.4)
p for trend		0.65		0.25		0.04		0.60

^*^ Least square means and p values for trend from linear regression. Adjusted for age at mammography (continuous), years of education (≤ 11, 12–14, 15+), number of pregnancies (nulliparous, 1,2,3,4, 5–10), age at first full-term pregnancy for parous women (<20, 20–24, 25–29, 30+), postmenopausal hormone therapy (never, past, current), body mass index (continuous).

When considering the intensity of physical activity, there was no association between light physical activity and percent mammographic density in any of the BMI strata (Table 3). However, there was a statistically significant inverse association between moderate physical activity and percent mammographic density among the overweight women. The percent mammographic density was 12.2% (11.4%- 13.0%) in the highest activity group (≤ 180 mins/week) whereas in the no activity group it was 14.6% (13.9% - 15.2%) (p for trend = 0.02). We also observed an inverse association between strenuous physical activity and percent mammographic density in the overweight women; this association was borderline statistically significant (p for trend = 0.06).

**Table 3:** Multivariate adjusted^*^mean (95%CI) of percent (%) mammographic density by light, moderate, strenuous physical activity (mins/week) for all women and stratified by BMI

VARIABLES	ALL		In strata of BMI					
			< 25		≥ 25-29		≥ 30	
	N = 2218		N = 1235		N = 732		N = 251	
**Light_activity (mins/week)**								
No activity	361	17.3 (16.4- 18.1)	189	23.4 (22.5- 24.3)	111	13.1 (12.3- 14.0)	61	8.4 (7.8- 9.0)
≤ 45**	384	17.7 (16.9- 18.4)	188	22.6 (21.8- 23.4)	143	13.8 (13.0- 14.7)	53	8.1 (7.5- 8.6)
46-100	507	18.4 (17.8- 19.0)	276	23.2 (22.5- 24.0)	175	13.8 (13.0- 14.5)	56	8.0 (7.4- 8.7)
101-180	450	18.2 (17.6- 18.9)	250	22.8 (22.0- 23.6)	156	13.6 (12.9- 14.3)	44	7.0 (6.2- 7.9)
>180	516	19.2 (18.1- 19.7)	332	23.2 (22.6- 23.9)	147	14.3 (13.6- 14.6)	37	7.0 (6.0- 8.2)
p for trend		0.45		0.79		0.54		0.27
**Moderate_activity (mins/week)**								
No activity	664	17.2 (16.7- 17.8)	341	21.4 (20.7- 22.0)	236	14.6 (13.9- 15.2)	87	8.5 (7.9- 9.0)
≤ 30	257	17.8 (16.9- 18.6)	130	22.1 (21.2- 23.1)	91	15.0 (14.1- 16.0)	36	8.5 (7.7- 9.2)
31- 45	457	18.3 (17.5- 19.0)	258	23.4 (22.6- 24.1)	142	13.3 (12.6- 14.1)	57	7.3 (6.7- 8.0)
46-100	414	18.6 (17.8- 19.4)	231	24.2 (23.4- 25.0)	138	13.0 (12.3- 13.8)	45	6.8 (6.0- 7.7)
101-180	426	19.8 (19.2- 20.4)	275	24.5 (23.8- 25.3)	125	12.2 (11.4- 13.0)	26	7.0 (6.2- 7.7)
p for trend		0.94		0.11		0.02		0.23
**Strenuous_activity (mins/week)**								
No activity	1404	17.4 (17.0- 17.8)	727	22.3 (21.8-22.7)	490	14.0 (13.5- 14.4)	187	7.5 (7.2- 7.9)
≤ 30	218	18.2 (17.2- 19.1)	118	22.6 (21.5-23.7)	72	13.7 (12.7- 14.8)	28	7.5 (6.7- 8.2)
31- 45	265	19.9 (19.0- 20.9)	174	24.5 (23.6- 25.4)	69	13.2 (12.2-14.2)	22	8.4 (6.9- 9.9)
46- 100	197	20.2 (19.3- 21.1)	119	24.9 (23.8- 26.0)	69	12.6 (11.5- 13.7)	9	9.6 (6.6- 12.6)
101-180	134	21.5 (20.5- 22.4)	97	25.2 (24.0- 26.3)	32	12.7 (11.0- 14.4)	5	10.9 (6.6- 15.3)
p for trend		0.80		0.83		0.06		0.37

^*^ Least square means and p values for trend from multiple linear regression modeling light, moderate and strenuous activity simultaneously. Adjusted for age at mammography (continuous), years of education (≤ 11, 12–14, 15+), number of pregnancies (nulliparous, 1,2,3,4, 5–10), age at first full-term pregnancy for parous women (<20, 20–24, 25–29, 30+), postmenopausal hormone therapy (never, past, current), body mass index (continuous).

** Women with ≤ 30 mins of light activity are included in the no activity group.

We also examined the relationship between the combination of moderate and strenuous physical activity and mammographic density (Table 4). The analysis was adjusted for light physical activity. There was no association between moderate/strenuous activity and MD overall but the association between activity and%MD differed by BMI. Consistent with the results mentioned above, we observed an inverse association between the combined moderate/strenuous physical activity and mammographic density in the overweight women.

**Table 4:** Multivariate adjusted^*^mean (95%CI) of percent (%) mammographic density by moderate + strenuous physical activity (mins/week) for all women combined and stratified by BMI

VARIABLES	ALL		In strata of BMI					
			< 25		≥ 25-29		≥ 30	
	N = 2218		N = 1235		N = 732		N = 251	
Moderate + strenuous activity (mins/week)								
No activity	566	16.9 (16.3- 17.5)	287	20.9 (20.2- 21.6)	200	14.8 (14.2- 15.5)	79	8.0 (7.5- 8.5)
≤ 30	193	17.2 (16.1- 18.2)	90	21.9 (20.8- 23.1)	73	14.9 (13.9- 16.0)	30	8.4 (7.6- 9.2)
31- 45	265	17.2 (16.2- 18.2)	139	22.9 (21.8- 24.0)	84	13.0 (12.0 - 14.1)	42	7.5 (6.7- 8.3)
46- 100	435	18.5 (17.8- 19.1)	238	23.2 (22.4- 23.9)	149	13.8 (13.1- 14.6)	48	7.3 (6.6- 8.0)
101-180	426	19.1 (18.4- 19.8)	254	24.0 (23.2- 24.8)	136	12.5 (11.7 -13.3)	36	7.7 (6.8- 8.7)
>180	333	20.6 (20.0- 21.3)	227	25.4 (24.6- 26.1)	90	12.1 ( 11.2- 13.0)	18	7.2 (5.8- 8.6)
p for trend		0.81		0.06		0.001		0.51

^*^ Least square means and p values for trend from linear regression. Adjusted for age at mammography (continuous), years of education (≤ 11, 12–14, 15+), number of pregnancies (nulliparous, 1,2,3,4, 5–10), age at first full-term pregnancy for parous women (<20, 20–24, 25–29, 30+), postmenopausal hormone therapy (never, past, current), body mass index (continuous), light physical activity.

The percent mammographic density was 12.1% (11.2%- 13.0%) in the highest activity group (> 180 mins/week) whereas in the no activity group it was 14.8% (14.2% - 15.5%) (p for trend = 0.001). However, in the normal weight women, there was a positive borderline significant association between moderate/strenuous activity and mammographic density with percent mammographic density of 25.4% (24.6%- 26.1%) in the highest group (> 180 mins/week), and 20.9% (20.2%- 21.6%) in the no activity group (p for trend = 0.06). Analyses were also conducted with absolute density as the outcome measure. The results were not different, from the abovementioned results (results not shown). We also conducted analysis by modeling intensity of physical activity (light, moderate, strenuous) as continuous variables separately without mutually adjusting for each other. The results were essentially the same (results not shown).

Finally we conducted analyses stratified by age and postmenopausal hormone therapy to determine if either variable modified the association between physical activity and mammographic density. There was no evidence of effect modification by either in all women combined or in any of the BMI strata (results not shown).

## Discussion

In this study total physical activity was unrelated to MD overall. However, in heavier women we found some evidence of an inverse association between total physical activity and mammographic density, and between moderate as well as strenuous physical activity and percent mammographic density.

Our overall finding of no statistically significant association between total physical activity and mammographic density is consistent with many other studies which reported no association between physical activity and percent mammographic density (Gram et al. 1999; Suijkerbuijk et al. 2006; Siozon et al. 2006; Peters et al. 2008; Conroy et al. 2010; Samimi et al. 2008; Reeves et al. 2007; Woolcott et al. 2010).

Our results are supportive of an inverse association between physical activity and mammographic density appearing in over-weight and obese women. Our results are consistent with results of two previous studies that stratified analyses by BMI. Both studies reported inverse associations between physical activity and mammographic density in heavier women (Irwin et al. 2006; Masala et al. 2009). One of these studies was a cross-sectional analysis of 474 participants who reported their usual physical activity a year prior to their diagnosis of breast cancer from two different centers in the US (Irwin et al. 2006). No statistically significant trends were observed between total physical activity or sports/recreational physical activity and dense breast area or percent mammographic density after adjustment for BMI as a continuous variable. However, when the analyses were stratified by BMI, statistically significant inverse associations were observed between physical activity and mammographic density among obese (BMI ≥ 30 kg/m^2^) postmenopausal women (Irwin et al. 2006). In a study of Italian women, there was an inverse association between physical activity among postmenopausal women that was more evident in the highest BMI tertile (≥ 26.5 ) (Masala et al. 2009).

Physical activity is only inversely associated with mammographic density in overweight/obese women. In other words, one could hypothesize that the effect of physical activity would be stronger in overweight women. A year long randomized controlled trial, reported that physical activity had a favorable effect on reducing circulating sex hormone concentrations among overweight postmenopausal women (McTiernan et al. 2004; Friedenreich 2011). Thus, if this effect is stronger in overweight than in normal weight women, it could explain a possible modifying effect of BMI on the association between physical activity and mammographic density. If true, this could suggest that physical activity is particularly beneficial in reducing breast cancer risk in heavy women (Woolcott et al. 2010).

A proposed mechanism explaining the association between physical activity and mammographic density is that physical activity may alter female sex steroid hormone levels that may result in reduced mammographic density. The effect of endogenous estrogens on mammographic density is however, not clear (Tamimi et al. 2005; Tamimi et al. 2007; Boyd et al. 2002b; Greendale et al. 2005; Bremnes et al. 2007). Three studies have examined the relationship between plasma levels of endogenous sex steroid hormones and mammographic density among postmenopausal women (Tamimi et al. 2005; Boyd et al. 2002b; 2005). Boyd et al. (2002b) found no positive association between levels of circulating free estradiol and mammographic density among 189 postmenopausal women, after adjusting for age and waist measurements. Among 520 women in the Nurses’ Health Study, Tamimi et al. observed an inverse association between estradiol and mammographic density which was no longer statistically significant after adjustment for BMI (Tamimi et al. 2005). In contrast, the Postmenopausal Estrogen – Progestin Interventions study found a positive, association between endogenous estradiol levels and mammographic density (Greendale et al. 2005). An Italian study of recently postmenopausal women also reported a positive association between mammographic density and endogenous estradiol (Hofvind et al. 2011).

Thus there is some, although not overwhelming evidence for an association between endogenous levels of estrogen and mammographic density. If there is such an association, then the question is whether estrogen levels could have a stronger affect in heavier than in lighter women. In the Alberta Physical Activity and Breast Cancer Prevention (ALPHA) Trial physical activity resulted in larger reduction in estrone levels in over-weight than in normal weight women (Tretli & Haldorsen 1990).

A recent systematic review of 33 cohort studies and 40 case–control studies published until Dec 2009, found that physical activity was associated with a reduction in risk of breast cancer (Lynch et al. 2011). According to these studies physically active women had on average 25% less risk of breast cancer as compared to the least active women. Recreational activity, regular activity sustained over life time, activity of moderate to vigorous intensity and that performed after menopause had the strongest association. Physical activity provides many health benefits, including weight loss and maintenance, improved insulin sensitivity, and improved lipid profile. Alterations in the metabolism of endogenous hormones such as insulin, sex hormones and levels of insulin-like growth factor (IGF)- I, and IGF-binding proteins (IGFBPs), may form the causal pathways linking excess weight and breast cancer risk (Bianchini et al. 2002).

The cross-sectional design of our study limited our ability to draw a temporal association. Another possible limitation of our study could have been that we had measurement of mammographic density at one point in time only, and thus were precluded from assessing the effect of physical activity on mammographic density over time. As we did not have information on household and occupational activity from our questionnaire, therefore we cannot comment if there is any association with these types of activity and mammographic density. We had to rely on the information about physical activity reported by the women themselves based on the questionnaire. Thus we cannot rule out the possibility of over reporting and possible misclassification of the physical activity variables.

A fundamental problem in making a causal interpretation of associations from observational data is the possibility that such associations are due to confounding. In our study, estimates were adjusted for important confounding factors including age, BMI, education, age at menarche, use of hormone therapy. We cannot exclude the possibility that our estimates could be residually confounded. In particular BMI, as the weight and height were self reported in our study it is likely that this would have caused misclassification for the BMI variable. Moreover, the assessment of adiposity by BMI rather than a more precise measures of body fat (e.g. DXA) may appear to be a limitation of the study, however, Woolcott *et al*. found anthropometric measurements are likely to be sufficient for adjustment of the association between mammographic density and breast cancer risk (Conroy et al. 2012).

There were several strengths of this study. The mammographic density was assessed by trained personnel using a validated computer assisted method. Readers had no knowledge of the physical activity and risk factor data, thus minimizing the chances of systematic error due to observation bias. Another strength of our study is that we used a continuous measure of mammographic density, which more accurately represents the relation with breast cancer risk (McCormack & Dos Santos 2006). Recall bias is unlikely to have been a problem in our study, since women typically do not know their mammographic density. In addition, not knowing their mammographic density the decision to participate in the study was independent of their knowledge, hence ruling out the possibility of a potential selection bias.

In conclusion, we found some evidence of an inverse between physical activity and mammographic density among women in the over-weight BMI stratum. However, our findings are limited to one BMI stratum and to total physical activity only, thus we cannot rule out the possibility that these findings could be due to chance. Further studies are needed to confirm any associations between physical activity (including other types household, occupational activity as well) and mammographic density, and in particular to better understand the confounding or possibly modifying effects of BMI.
